# DPP-4 inhibition has no acute effect on BNP and its N-terminal pro-hormone measured by commercial immune-assays. A randomized cross-over trial in patients with type 2 diabetes

**DOI:** 10.1186/s12933-017-0507-9

**Published:** 2017-02-10

**Authors:** Gian Paolo Fadini, Benedetta Maria Bonora, Mattia Albiero, Martina Zaninotto, Mario Plebani, Angelo Avogaro

**Affiliations:** Department of Medicine, University of Padova, Via Giustiniani,2, 35128 Padua, Italy

**Keywords:** Heart failure, Enzyme, Proteases, Linagliptin, Kidney disease

## Abstract

**Background:**

Use of dipeptidyl peptidase-4 inhibitors (DPP4-i) for the treatment of type 2 diabetes (T2D) has been associated with a possible increase in the risk for heart failure (HF). B-type natriuretic peptide (BNP), which is both a biomarker of HF and a hemodynamically active hormone, is a substrate of DPP-4. We herein tested the acute effects of the DPP-4i linagliptin on BNP and NT-proBNP in a cross-over placebo-controlled trial in patients with T2D with and without chronic kidney disease (CKD).

**Methods:**

B-type natriuretic peptide and NT-proBNP were measured using commercially available clinical-grade immune-assays at baseline and at the end of a 4-day treatment with placebo and linagliptin. Changes from baseline during each treatment arm, as well as placebo-subtracted effects of linagliptin on BNP and NT-proBNP were calculated.

**Results:**

46 patients completed the study, 18 of whom were affected by CKD. Baseline BNP and NT-proBNP levels increased with age, were elevated in CKD patients, and inversely correlated with estimated glomerular filtration rate. No significant change was detected in BNP and NT-proBNP levels after treatment with linagliptin or placebo in patients with or without CKD. Only in CKD patients the placebo-subtracted effect of linagliptin indicated a significant reduction in NT-proBNP levels, but this finding was not statistically robust.

**Conclusions:**

Acute treatment with a DPP-4i exerts no clinically-meaningful effects on BNP and NT-proBNP. As routinely used immunoassays do not discriminate between intact/active and cleaved BNP, these data cannot rule out an effect of DPP-4i on HF pathophysiology.

*Trial registration* NCT01617824

## Background

Dipeptidyl peptidase-4 inhibitors (DPP-4i) are routinely used for the treatment of type 2 diabetes (T2D). Their glucose-lowering activity results from inhibition of the enzymatic cleavage of glucagon-like pepide (GLP)-1 and glucose insulinotropic peptide (GIP) by DPP-4 [1]. DPP-4 has several other physiological substrates, including cytokines, chemokines, and neuro-hormones, providing the biological basis for non-glycemic effects of DPP-4i [2]. However, comparatively fewer peptides have been identified as endogenous physiological substrates in vivo [3].

Concerns have been raised on the possibility that DPP-4i increase the risk of heart failure (HF), but the mechanisms are largely unknown [4, 5]. Intact B-type natriuretic peptide (BNP_1–32_) is cleaved by DPP-4, generating BNP_3–32_ [6]. BNP, which is produced by cardiomyocytes in response to hemodynamic stress and neuro-hormonal stimulation, is a clinical-grade biomarker of HF [7]. In turn, BNP is involved in the pathophysiology of HF, as it induces vasodilation and natriuresis, thereby antagonizing the effects of angiotensin-II [8, 9]. BNP concentrations are reduced in people with obesity, insulin resistance, and diabetes, and this deficiency may contribute to their cardiovascular risk [10]. Thus DPP-4i may exert beneficial effects on cardiac function, by increasing the proportion of intact/active BNP_1–32_ versus cleaved BNP_3–32_, in addition to GLP-1 mediated cardioprotection [11].

**Fig. 1:**
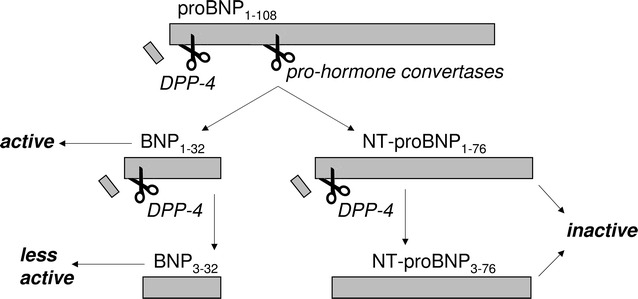
Sequential cleavage of proBNP to originate BNP, NT-proBNP and their by-products. Biological activity on cardiac function is reported. DPP-4 can cleave the 2 N-terminal residues of proBNP, BNP, and NT-proBNP, generating inactive or less active peptides

BNP_1–32_ derives from proBNP (108 amino-acid) after removal of an N-terminal (NT) fragment of 76 amino-acids by pro-hormone convertases [12]. Although BNP and NT-proBNP are released at equimolar concentrations, the half-life of the NT-proBNP in the circulation is longer, resulting in higher concentrations. In patients with diabetes, despite a possible reduction of BNP, the clinical predictive capacity of NT-proBNP has been shown to be preserved [13, 14]. Interestingly, also proBNP_1–108_ and NT-proBNP_1–76_ are candidate substrates of the enzymatic activity of DPP-4, as they have proline in the second N-terminal position, where the exopeptidase activity of DPP-4 locates. Commercially available immuno-assays are presumably unable to distinguish between BNP_1–32_ and BNP_3–32_ [15], and they may even detect proBNP_1–108/3–108_, but not NT-proBNP [16]. As compared to BNP_1–32_, truncated BNP_3–32_ appears to have equal cGMP activating properties in vitro [17], but lower activity in vivo [18], probably because of a higher susceptibility to degradation by other peptidases. Specificity of NT-proBNP immunoassays is unknown, but epitopes recognized by monoclonal antibodies do not appear to span the first 2 N-terminal residues. Based on these considerations, the net effect of a DPP-4i therapy on diagnostic BNP and NT-proBNP determinations is unpredictable (Fig. 1).

Available data on the effects of DPP-4i on proBNP-derived peptides in T2D mostly come from large clinical trials wherein NT-proBNP levels were measured years after therapy with a DPP-4i or placebo [19, 20]. Rather, if DPP-4i has direct effects on proBNP processing, this should be detectable within a short time frame. To address the acute effects of DPP-4i on BNP and NT-proBNP, we used samples from a placebo-controlled cross-over trial testing the effects of a 4-day therapy with the DPP-4i inhibitor linagliptin on humoral factors [21]. Differently from other DPP-4i, linagliptin has no renal excretion, and is thereby particularly suitable for the treatment of patients with chronic kidney disease (CKD) [22, 23], which is a major risk factor for HF [24].

## Methods

### Study design

The NCT01617824 was a randomized, single-blind, placebo-controlled, cross-over study designed to test the acute effects of the DPP-4 inhibitor linagliptin on cytokines, hormones, and inflammatory mediators. The primary study results have been published before [21]. Briefly, T2D patients with or without CKD, received a 4-day treatment with linagliptin 5 mg and placebo in a random order with a 14-day wash-out period. Before and at the 5th day of each treatment period, fasting blood samples were drawn. Aliquots of EDTA and heparin plasma were separated and stored at −80 °C until analyses. CKD was defined as an estimated glomerular filtration rate (eGFR) of less than 60 ml/min/1.73 mq, based on the CKD-EPI formula [25] and graded according to the Kidney Disease Outcomes Quality Initiative (KDOQI) [26]. With this design, we were able to detect significant changes in DPP-4 activity, levels of intact (uncleaved) GLP-1 and SDF-1α, along with an increase in CD34^+^KDR^+^ cells, reflecting a biological consequence of elevated SDF-1α [21], an effect that was observed also for saxagliptin [27]. For this study, untouched frozen aliquots of plasma were thawed and used for the quantification of BNP and NT-proBNP.

### Analytical methods

B-type natriuretic peptide was quantified in EDTA plasma using a chemiluminescent, microparticle-capture immunoassay (Abbott Diagnostics kit, #8K28) on the modular automated ARCHITECT iSystem platform. The range of plasma BNP concentrations revealed using this assay is 10–5000 pg/ml. The coefficient of variation (CV), as reported by the manufacturer, is <5%. This BNP assay results in no cross-reactivity with ANP, Angiotensin-I, Angiotensin-II, Angiotensin-III, CNP, and NT-proBNP.

NT-proBNP was quantified in heparinised plasma using a solid-phase two-site chemiluminescent immunometric assay (Siemens IMMULITE 1000 Turbo). The sensitivity of this assay is 15 pg/ml, with a reportable range up to 35,000 pg/ml. The CV reported by the manufacturer is 9%. No cross-reactivity has been detected with ANP, NT-proANP, BNP, CNP, Adrenomedullin, Angiotensin-I, Angiotensin-II, Angiotensin-III, Endothelin, Renin, Urodilatin, and Arg-Vasopressin.

### Statistical analysis

Data are expressed as mean ± standard error if normal or as median (interquartile range) if not normal. Normality was checked using the Shapiro–Wilk test and non-normal variables were log-transformed before analysis. Within-group changes in continuous variables were analyzed using the paired two-tail Student’s *t* test. For each patients in each group of treatment order, we calculated the effect of placebo, the effect of linagliptin, and the placebo-subtracted effect of linagliptin. The generalized linear model (GLM) was used to analyze the effect of treatment and order by the cross-over design. Statistical significance was accepted at p < 0.05 and SPSS version 22.0 was used.

Sample size was originally chosen to achieve a 80% power to detect a significant difference in the primary end-point (a difference in circulating CD34^+^KDR^+^ cells), which was fully satisfied. Based on within-patients standard deviations of 37% for BNP (26 pg/ml) and 40% for NT-proBNP (227 pg/ml), we calculated a priori that this study had 80% power to detect a treatment difference at a two-sided 0.05 significance level, if the true difference between treatments was 22% for BNP (15 pg/ml) and 24% for NT-proBNP (136 pg/ml).

## Results

### Characteristics of study patients

A total of 46 patients completed the study. Detailed baseline clinical characteristics of the participants have been reported previously [21] and are herein summarized in Table 1. There was no difference between patients randomized to the placebo-linagliptin (n = 22) or the linagliptin-placebo (n = 24) treatment order. No mild or severe averse event was reported during treatment or wash-out and no change in fasting metabolic variables (glucose, triglycerides and fatty acids) was observed [21].

**Table 1:** Baseline characteristics of study patients

Variable	All patients
Number	46
Age, years	63.7 ± 1.3
Sex male, %	71.7
Body mass index, kg/m^2^	31.1 ± 0.7
Waist, cm	105.4 ± 2.2
HbA1c, % (mmol/mol)	7.6 ± 0.2 (60 ± 2)
Risk factors	
Smoking habit, %	13.0
Hypertension, %	89.1
Total cholesterol, mg/dl	165.3 ± 5.5
HDL cholesterol, mg/dl	49.9 ± 2.2
LDL cholesterol, mg/dl	91.6 ± 5.1
Triglycerides, mg/dl	119.2 ± 8.5
Albumin/creatinine ratio (mg/g)	129.5 ± 44.1
Creatinine, mg/dl	1.11 ± 0.06
eGFR, ml/min/1.73 mq	75.5 ± 3.9
Complications	
Retinopathy, %	28.2
Neuropathy, %	17.9
Coronary artery disease, %	30.4
Peripheral arterial disease, %	21.7
Cerebrovascular disease, %	47.8
Medications	
Metformin, %	65.2
Sulphonylurea, %	6.5
Repaglinide, %	4.3
Pioglitazone, %	6.5
Insulin, %	43.4
ACE inhibitors/ARBs, %	76.1
Other anti-hypertensives, %	78.2
Statin, %	80.4
Anti-platelet agents, %	56.5

Data are presented as mean ± standard error, or as percentage, where appropriate. More details can be found in [21]

### Baseline values of BNP and NT-proBNP

As the distributions of BNP and NT-proBNP were highly skewed, data are presented as median (IQR) and values were log-transformed before statistical testing.

The median baseline plasma BNP level was 20.4 pg/ml (IQR 10.0–43.3). BNP was below threshold (10 pg/ml) in n = 15 patients (32.6%) and was above the decisional cut-off (100 pg/ml) [28] in 6 patients (13.0%). BNP levels increased with age (r = 0.40; p = 0.003), were higher in patients with CKD than in those without (43.1 [IQR 22.1–98.5] versus 12.5 [IQR 10.0–23.0] pg/ml; p = 0.0022) and were inversely correlated with eGFR (r = −0.45; p < 0.001).

The median baseline NT-proBNP level was 101.0 pg/ml (IQR 35.3–314.8) and n = 5 patients (10.9% had values above the decisional cut-off (900 pg/ml) [28]. NT-proBNP levels increased with age (r = 0.52; p < 0.001), were higher in patients with CKD than in those without (238.5 [IQR 115.0–554.8] versus 44.0 [IQR 24.3–101.0] pg/ml; p < 0.001) and were inversely correlated with eGFR (r = −0.50; p < 0.001).

Levels of BNP and NT-proBNP were highly correlated (r = 0.94).

### Effects of DPP-4 inhibition on BNP and NT-proBNP

**Fig. 2:**
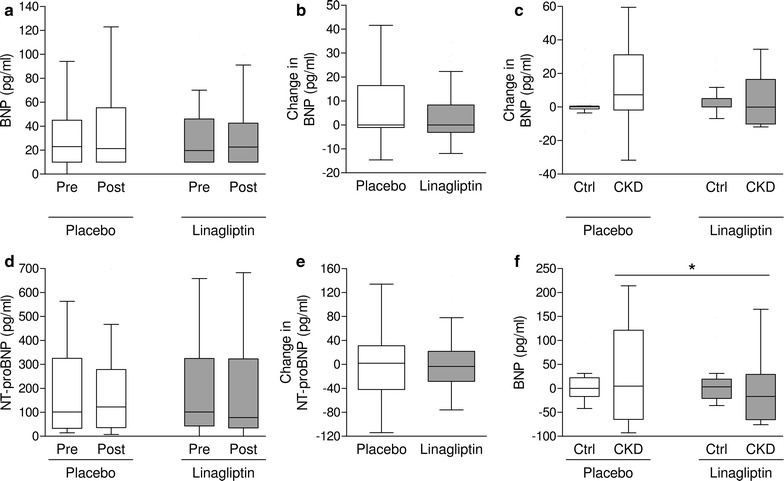
BNP and NT-proBNP levels during treatment with placebo and linagliptin. Data are presented as baseline (pre) and end-of-treatment (post) values (**a**, **d**), and change from baseline (**b**, **e**) during placebo or linagliptin. **c**, **f** Show changes from baseline in BNP and NT-proBNP, respectively, in patients with (n = 18) CKD and in those without (n = 28; Ctrl). *p<0.05. The *box plot* shows median and IQR, whereas *whiskers* indicate Tukey range

Overall, no significant change versus baseline was observed in BNP and NT-proBNP levels after treatment with linagliptin or placebo (Fig. 2). The placebo-subtracted effect of linagliptin on BNP and NT-proBNP were 0.0 pg/ml (IRQ −19.0 to 1.7) and −19.5 pg/ml (IQR −62.3 to 19.3), respectively. In patients with CKD (n = 18) the change of NT-proBNP during linagliptin treatment was not significant (−17.0; IRQ −59.3 to 23.0 pg/ml), but was significantly different from the change during placebo (4.5; IQR −57.8 to 109.3 pg/ml; p = 0.022). This resulted in a significant placebo-subtracted reduction in NT-proBNP attributable to linagliptin treatment (Table 2; Fig. 2). The post hoc power of the study to detect this finding was calculated to be <20%. Two patients with CKD had a previous history of HF, but placebo-subtracted changes of BNP (−72.7 and +34.0 pg/ml) or NT-proBNP (−42 and +216 pg/ml) were inconsistent with an effect of DPP-4i.

**Table 2:** BNP and NT-proBNP levels, expressed as median (IQR) during treatment with placebo or linagliptin

	Placebo			Linagliptin			Placebo-subtracted change
	Pre	Post	Change	Pre	Post	Change	
BNP							
All	22.9 (10.0–42.8)	21.3 (10.0–52.6)	0.0 (−0.9 to 15.2)	19.6 (10.0–44.3)	22.6 (10.0–39.3)	0.0 (−2.7 to 7.8)	0.0 (−19.0 to 1.7)
No CKD	11.4 (10.0–23.3)	11.9 (10.0–29.5)	0.0 (−0.9 to 0.3)	10.6 (10.0–21.8)	12.5 (10.0–27.4)	0.0 (0.0–4.6)	0.0 (−1.0 to 1.2)
CKD	38.5 (26.8–80.3)	56.3 (24.6–107.1)	7.3 (−0.7 to 26.0)	44.2 (20.3–129.0)	37.4 (25.0–78.9)	0.0 (−9.5 to 12.1)	−17.6 (−46.8 to 0.8)
NT-proBNP							
All	101.0 (36.5–314.5)	122.0 (38.0–267.0)	2.0 (−32.0 to 30.5)	101.0 (44.0–299.0)	78.0 (36.0–272.0)	−3.5 (−27.5 to 19.3)	−19.5 (−62.3 to 19.3)
No CKD	46.0 (26.0–101.0)	57.0 (20.0–167.0)	0.0 (−12.0 to 20.0)	51.0 (28.5–121.5)	41.0 (26.5–99.0)	3.0 (−17.5 to 17.3)	−2.5 (−35.0 to 15.8)
CKD	218.5 (108.8–554.8)	261.0 (130.0–696.5)	4.5 (−57.8 to 109.3)	238.5 (107.5–611.5)	184.0 (109.0–483.0)	−17.0* (−59.3 to 23.0)	−50.0^#^ (−101.5 to 18.0)

* Significantly different from placebo treatment (p < 0.05 at paired *t* test on log-transformed data or Mann–Whitney test)

^#^ Significantly different from zero

No carry-over effect was noted for both BNP and NT-proBNP. No correlation was detected between BNP or NT-proBNP and DPP-4 activity, nor between change in BNP or NT-proBNP and change in DPP-4 activity.

## Discussion

We show that therapy with a DPP-4i has no acute effects on BNP and NT-proBNP levels measured with routine diagnostic immuno-assays. This study was not designed to test the acute effects of DPP-4i on cardiac function, but our findings re-assure on the safety of DPP-4i concerning diagnosis and prognostic evaluation of HF.

The clinical relevance of the interplay between DPP-4i and BNP/NT-proBNP levels has emerged after publication of the results of SAVOR-TIMI trial, wherein patients treated with the DPP-4i saxagliptin exhibited a significant 27% excess risk of hospitalization for HF compared to placebo [19, 29]. Meta-analyses of randomized controlled trials were unable to rule out the concern that DPP-4i therapy may favour HF [4, 5]. Real-world data did not confirm an association between DPP-4i and hospitalization for HF [30, 31], nor show adverse prognosis in HF patients treated with DPP-4i [32, 33]. Furthermore, the eventual mechanisms remain elusive. In the SAVOR-TIMI trial, the risk of HF associated with saxagliptin therapy was almost exclusively observed in patients with a baseline NT-proBNP level within the most elevated quartile [19]. During a follow-up of about 2 years, NT-proBNP levels increased in both the placebo and saxagliptin group, but the increase was slightly blunted by saxagliptin [19]. The relevance of this finding is limited because exceeding HF cases in saxagliptin-treated patients were observed only in the first 6 months of therapy [19]. In another placebo-controlled trial conducted on T2D patients after an acute coronary event, the DPP-4i alogliptin was associated with a non-significant increase in the risk of hospitalization for HF [34]. During an average 1.5 year follow-up, NT-proBNP concentrations decreased significantly and similarly in the two groups [20]. Our study shows for the first time that BNP and NT-proBNP are not directly affected by DPP-4i because no acute effect was detected. This suggests that the observed changes in NT-proBNP over the long run most likely reflect the natural history of HF, rather than effects of therapy.

Chronic kidney disease is a one of the strongest risk factors for HF [24]. As BNP and NT-proBNP levels were higher in CKD patients, the amplitude of their excursions after DPP-4i or placebo was also larger. Although consistent with a study showing that linagliptin decreased BNP in an experimental model of uremic cardiomyopathy [35], the modest placebo-subtracted effect of DPP-4i on NT-proBNP reduction we observe in CKD patients had very low statistical power, was no longer significant after adjusting for multiple testing, and is unlikely to be of any clinical meaning, as NT-proBNP is not biologically active.

The present study has limitations inherent to the small sample size, the fact that a minority of patients had CKD, thereby lowering power in this subgroup, and the lack of data in patients with decompensated HF. Finally, more subtle changes in BNP and NT-proBNP induced by DPP-4i may have been missed, since the study was powered for a minimal detectable change of 22 and 24%, respectively. Since clinical-grade commercially available immuno-assays do not distinguish the intact and cleaved forms of BNP and NT-proBNP, our data provide no clear indication of whether DPP-4i interferes with the in vivo processing of the two peptides, and whether it intervenes in the pathophysiology of HF. However, any eventual significant change in the relative proportion of BNP_1–32_ and BNP_3–32_ or in the proportion of NT-proBNP_1–76_ and NT-proBNP_3–76_ induced by DPP-4i may nonetheless result in modifications of immune-reactive (total) BNP and NT-proBNP levels, respectively. This has been shown for GLP-1 and SDF-1α [21], possibly reflecting compensatory secretion and/or changes in sequential cleavage by different peptidases. Furthermore, experimental studies suggest that linagliptin exerts favourable effects on ischemia–reperfusion injury [36], which in the long-term can translate into protection from HF.

## Conclusion

Although exact discrimination of the various proBNP-derived peptides will require sophisticated, time consuming and costly mass spectrometric approaches [15], data obtained with diagnostic assays indicate that DPP-4i has no clinically appreciable effects on BNP and NT-proBNP. Further studies will be needed to dissect whether DPP-4i interferes with the biological action of BNP and whether this is linked to HF risk in patients with T2D.

## Abbreviations

ANP: atrial natriuretic peptide
BMI: body mass index
BNP: B-type natriuretic peptide
CKD: chronic kidney disease
CNP: cardiac natriuretic peptide
DPP-4: dipeptidyl peptidase-4
EDTA: ethilendiaminoacetic acid
eGFR: estimated glomerular filtration rate
GIP: glucose-insulinotropic peptide
GLM: generalized linear model
GLP-1: glucagon-like peptide 1
HF: heart failure
IQR: interquartile range
KDOQI: kidney disease outcomes quality initiative
NT: N-terminal
SDF: stormal derived factor
T2D: type 2 diabetes
